# Case Report: Jejunoileal Atresia With Persistent Poor Bowel Function Can Occur After Surgical Correction for Hirschsprung Disease

**DOI:** 10.3389/fped.2022.907179

**Published:** 2022-05-20

**Authors:** Bassam N. AlBassam, Ahmad A. Al-Shammari, Saleh A. AlQahtani, Elham Hassan

**Affiliations:** ^1^Department of Pediatrics, College of Medicine, Imam Abdulrahman bin Faisal University, Dammam, Saudi Arabia; ^2^Department of Paediatrics, King Fahd Hospital of the University, Al-Khobar, Saudi Arabia

**Keywords:** pediatric, ileal atresia, Hirschsprung disease, immunodeficiency, Saudi Arabia

## Abstract

Jejunoileal atresia (JIA) is one of the common etiologies of intestinal obtrusion in neonates. However, cases of concomitant ileal atresia and Hirschsprung disease (HD) rarely occur. We report the case of a male infant who had JIA concomitantly with HD that was re-anastomosed. The patient underwent an exploratory laparotomy to resect the dilated terminal ileum. Subsequently, owing to a significantly dilated proximal bowel, he underwent a second exploratory laparotomy. However, he continued to have feeding intolerance postoperatively. He had colonic aganglionosis and was diagnosed with HD. A third laparotomy was then performed. Additionally, he had recurrent episodes of gram-negative bacteremia, especially *candida parapsilosis fungemia*, despite receiving antibiotics and antifungal, and there were no identifiable underlying genetic or immunological causes. Finally, the patient had recurrent episodes of hypoglycemia, central hypothyroidism, and multiple organ failure and died at the age of 7 months. The concomitant ileal atresia and HD was thought to be due to a common intrauterine vascular accident, together with loss of bowel, thereby acting as a barrier for the caudal migration of neuromeric cells and leading to colonic aganglionosis. In this case, ileal atresia was associated with colonic aganglionosis, central hypothyroidism, and persistent bacteremia, which is a unique finding. In cases of JIA, persistent poor bowel function after surgical correction of concomitant HD should be considered.

## Introduction

Intestinal obstruction due to ileal and jejunal atresia, described together as jejunoileal atresia (JIA), is one of the common etiologies of intestinal obtrusion in neonates (1). The incidence of intestinal obtrusion ranges from 1 in 5,000 to 1 in 14,000 live births, and occurs equally in men and women (1–3). Additionally, JIA is associated with other concomitant malformations, such as cardiac anomalies, gastroschisis, cystic fibrosis, and other conditions (1, 4, 5). Concomitant ileal atresia and Hirschsprung disease (HD) is an extremely rare entity, with an incidence ranging from 0.25 to 0.8% (4).

In this study, we describe a pediatric case of JIA concomitant with HD that was re-anastomosed. Our case indicated that JIA was associated with persistent poor bowel function after surgical correction of concomitant HD. Thus far, no study has reported this finding. Moreover, the present study describes a unique combination of ileal atresia associated with colonic aganglionosis, central hypothyroidism, and persistent bacteremia, with no identifiable underlying genetic or immunological causes.

## Case Description

The patient was a 7-month-old Pakistani boy who was born in June 2021 at term gestation, with a birth weight of 2.7 kg resulting from an uneventful pregnancy and delivery history. The next day, he and his mother were discharged from the hospital in good clinical condition. According to the discharge summary, he passed meconium and urine before discharge, and had no reported concerns apart from hypospadias. There was no consanguinity between his parents, and his father and mother had good health. There was no known family history of gastrointestinal or congenital anomalies. He has three older siblings, and all are in good health.

At the age of 7 days, the patient was brought to the emergency room of King Fahd Hospital of the University, Eastern Province, Saudi Arabia with a history of non-projectile vomiting that started after discharge from the hospital. He vomited a small amount of milk for 3 days, which then progressed to a larger amount of bilious non-bloody emesis at a frequency of up to seven to eight times a day. The mother also reported that the patient had not passed stool at home since discharge, with increasing abdominal distension and oliguria for 3 days before presentation. He was on breastfeeding and formula feeding. Physical examination findings indicated an ill-looking infant who was conscious, alert, and hypoactive with moderate-to-severe signs of dehydration and scleral jaundice. He was febrile and had tachycardia with a normal-for-age blood pressure and respiratory rate. Growth parameters according to the World Health Organization chart were as follows: weight, 2.3 kg below the third centile for age and sex; length, 46 cm below the third centile for age and sex; and head circumference, 34.5 cm between the 50th and 15th centile for age and sex. Anterior fontanelle was open and at level, and he was moving his limbs freely. Abdominal examination findings revealed a shiny distended abdomen that was soft and lax with no tenderness or hepatosplenomegaly. Percussion revealed hyperresonance, and bowel sound was present on auscultation. Additionally, rectal examination findings revealed an empty rectum. The remaining findings of the physical examination were unremarkable.

Laboratory investigations upon admission included a septic workup that revealed high levels of C-reactive protein and procalcitonin, acute kidney injury with a picture of pre-renal azotemia with oliguria, and negative blood and urine cultures. Abdominal radiography results indicated a gaseous distended bowel and hemivertebral bodies at the thoracic spine. Because of bowel obstruction, the patient underwent a small bowel follow-through study, which revealed that diffused dilated gas filled the bowel loops, with no evidence of malrotation or volvulus at the time of the examination, suggestive of distal bowel obstruction. Barium enema findings indicated the presence of filling defects in the large colon, which was small in caliber with no contrast agent passing through the cecum or within the small bowel, suggestive of ileal atresia as the most likely diagnosis. Anomaly scans included an echocardiogram that revealed normal cardiac structure and function, apart from patent foramen ovale; a renal ultrasound, which revealed the presence of both kidneys with no associated anomalies; and a metastatic bone survey, which did not show skeletal deformities other than thoracic hemivertebral bodies.

The patient was admitted to the Pediatric Intensive Care Unit (PICU) of our institution for closer observation and stabilization. On the next day of admission, he underwent exploratory laparotomy through a right upper transverse incision, which found an adherent ascending and transverse colon with an unusually centrally located colon that was non-rotated. The atritic point was 5 cm proximal to the ileocecal valve, and the terminal ileum was dilated for approximately 10 cm proximal to that. The dilated portion was resected. Ileocolic anastomosis was performed, and the leak test showed no leaks. In addition, biopsy showed the absence of ganglion cells in the cecum and appendix. A few dysplastic ganglion cells along with prominent nerves were seen in the ascending colon.

For 6 weeks after the first operation, the patient did not have any bowel movements, even though the gastrointestinal contrast study revealed patent anastomosis but with a significantly dilated proximal bowel. A second exploratory laparotomy was performed, and 160 cm of hugely distended small bowel proximal to the ileocolic anastomosis was resected in addition to a microcolon situated in the right colic gutter. There was also 30 cm of normal caliber bowel distal to the ligament of Treitz. The ileocolic anastomosis was dismantled, enteroplasty was performed at the antimesenteric site of the small intestine, and ileocolic anastomosis was conducted. In addition, another biopsy revealed the presence of ganglion cells throughout the resected ileocolic anastomosis segment and anti-mesenteric border.

Owing to concerns regarding HD, a 1-cm strip biopsy was taken from the posterior rectal wall cranial to the level of the dentate line. The patient continued to experience significant small intestinal dilatation, and a small amount of bowel contents were passed through the rectum. The pathology report was consistent with HD. Based on the rectal biopsy results, a decision was made to proceed with a third laparotomy for the creation of an enterostomy. Extensive adhesions were found between the bowel loops and liver, which made it difficult to create an enterostomy at the site of the previous enterocolic anastomosis, and a new one was created more proximally. A distal mucous fistula was made proximal to the ileocolic anastomosis and brought out at the end of the wound. Colon biopsies revealed the presence of nerve hypertrophy and no ganglion cells, while the ileocolic anastomosis biopsy showed no nerve hypertrophy and a few dysplastic ganglion cells, and more proximally, the fistula edge biopsy showed ganglion cells and no nerve hypertrophy.

Afterward, the patient had recurrent episodes of gram-negative bacteremia, despite receiving treatment with appropriate antibiotics based on the extent of sensitivity in multiple cultures. He also had an episode of *candida parapsilosis fungemia*. Based on these infections, a suspected diagnosis of immunodeficiency, such as chronic granulomatous disease, was raised. An immunologic workup was performed in accordance with ongoing sepsis. A dose of intravenous immunoglobulin was administered, but there was no significant change in the patient’s clinical status. Findings of chromosomal microarray, primary immunodeficiency panel, and whole-exome sequencing (WES) were all unremarkable. The patient had recurrent episodes of hypoglycemia and was found to have central hypothyroidism. The patient subsequently developed multiple organ failure secondary to septic shock and died at the age of 7 months.

Informed consent was obtained from the patient’s parents.

## Discussion

Intestinal atresia is a congenital disease characterized by complete occlusion of the intestinal lumen (6). Causes of JIA have been attributed to intrauterine vascular events involving the mesenteric vessel branches of the midgut, leading to bowel necrosis and resorption (1, 4). Intestinal atresia has been classified into four types based on the Grosfeld classification system (Table 1). According to the criteria, our case would be classified as Type IIIa (1).

**TABLE 1:** Grosfeld classification of ileal atresia.

Type	Description
I	Internal membrane of the intestinal lumen that blocks the intestine with serosa continuity and causes no mesenteric defect.
II	Proximal and distal blind ends connected with a fibrous cord of atretic bowel with serosal discontinuity.
IIIa	Proximal and distal blind ends with a V-shaped mesenteric defect.
b	Blinded bowel distal to the atresia is wrapped around its collateral blood supply with a large V-shaped mesenteric defect, described as Apple peel or Christmas tree atresia.
IV	Multiple atresias as a short ileal segment coiled around the ileocolic artery.

Approximately one third to one half of JIA cases are diagnosed by routine prenatal ultrasound examination. Perinatal history of feeding intolerance, onset of abdominal distension, bilious or non-bilious vomitus, and failure to pass meconium are usually the first signs after birth that might indicate intestinal obstruction. The diagnosis is then made with radiological studies, including abdominal plain radiography, small bowel meal/follow-through and barium enema examinations (1, 3, 6). Other studies such as echocardiography, renal ultrasound, and rectal biopsy may be needed to rule out other concomitant malformations and associated diseases (1).

The resection of atretic and proximal dilated bowel with primary end-to-end anastomosis is the most common treatment option for JIA, although early studies on JIA showed high mortality rates (1, 6). This is thought to be due to functional obstruction resulting from defective peristalsis after anastomosis. More recent studies have shown improved survival, owing to advancements in surgical techniques, care at the intensive care unit, and nutritional support (1).

In contrast to JIA, which is thought to be due to a vascular defect in the intrauterine period, HD occurs due to the absence of ganglion cells in the myenteric (Auerbach) and submucosal (Meissner) plexuses, which is regarded as a neurogenic origin for intestinal obstruction (7, 8). Concomitant HD with ileal atresia can be explained through a possible common intrauterine vascular accident with loss of bowel that, in turn, may act as a barrier for neuromeric cells caudal migration, leading to colonic aganglionosis (4, 9). HD is reported to occur in 1 in 5,000 newborns (2). In case of concomitant JIA and HD, the patient would have non-resolving abdominal distension after primary small bowel surgical end-to-end anastomosis (1, 2). The same result was observed in our patient following the end-to-end anastomosis. Therefore, intestinal biopsies should be considered with the first surgical procedure of such JIA cases in which histological examination of ganglion cells will confirm the rare concomitant association (7).

Whole-exome sequencing is a useful tool for the diagnosis of hereditary diseases, especially after more specific genetic studies have ruled out more common causes. With regard to JIA, there is no well-known specific gene association (5, 10). However, a mutation in the *Tetratricopeptide Repeat Domain 7A* (*TTC7A*) gene has been associated with intestinal atresia combined with immunodeficiency (10). Because of the recurrent attacks of sepsis and persistent bacteremia in our patient, there was a high suspicion for immunodeficiency. However, with a negative primary immunodeficiency panel and an unremarkable WES result, the suspicion of a primary immunodeficiency was significantly reduced.

In conclusion, with persistent poor bowel function after surgical correction of JIA, we advise ruling out concomitant HD using rectal biopsy prior to surgical intervention. In addition, the occurrence of ileal atresia and frequent attacks of sepsis primary, immunodeficiency needs to be appropriately investigated and ruled out. Future research is needed to identify the underlying causes of these concomitant conditions.

## Data Availability Statement

The original contributions presented in the study are included in the article/supplementary material, further inquiries can be directed to the corresponding author.

## Author Contributions

AA-S and BA reviewed the literature. AA-S wrote the introduction and discussion. BA and SA reviewed the discussion. AA-S wrote the first draft of the manuscript. BA, SA, and EH read and approved the final version of the manuscript. All authors contributed to the article and approved the submitted version.

## Conflict of Interest

The authors declare that the research was conducted in the absence of any commercial or financial relationships that could be construed as a potential conflict of interest.

## Publisher’s Note

All claims expressed in this article are solely those of the authors and do not necessarily represent those of their affiliated organizations, or those of the publisher, the editors and the reviewers. Any product that may be evaluated in this article, or claim that may be made by its manufacturer, is not guaranteed or endorsed by the publisher.
